# Supplementary material for: A Likelihood Approach for Real-Time Calibration of Stochastic Compartmental Epidemic Models
Source: PLoS Comput Biol. 2017 Jan 17;13(1):e1005257. doi: 10.1371/journal.pcbi.1005257 (PMC5240920; doi:10.1371/journal.pcbi.1005257)
Supplement: S1 File — (TAR.GZ) [file pcbi.1005257.s014.tar.gz › HSPH_Online-SI-Revision/EnKF07/n100-mild/n100-mild_PF2_EnKF-summary2.pdf]

peak -8	3w spec	3w cum	5w spec	5w cum	end	3w spec LOG	3w cum LOG	5w spec LOG	5w cum LOG	end LOG
mean	1147.1	3311.4	119.3	684.3	80.8	1.	1.	1.5	0.7	0.6
median	280.5	1902.3	93.3	506.1	84.3	0.9	1.1	1.4	0.7	0.4
quantile .1	89.1	94.8	71.1	82.4	58.7	0.5	0.2	0.6	0.3	0.3
quantile .9	1971.5	7496.1	180.	1316.	95.8	1.5	1.7	2.5	0.3	1.1
26	18	27	14	37						

peak -4	3w spec	3w cum	5w spec	5w cum	end	3w spec LOG	3w cum LOG	5w spec LOG	5w cum LOG	end LOG
mean	92.2	135.4	91.7	93.1	84.4	1.5	0.6	2.2	0.7	0.8
median	95.8	106.2	93.6	92.5	85.6	1.4	0.6	2.3	0.8	0.9
quantile .1	75.2	76.	78.5	74.5	65.6	0.4	0.4	1.	0.3	0.3
quantile .9	105.8	255.9	98.4	111.4	96.6	2.3	0.8	3.	0.3	1.2
13	28	3	21	22						

peak	3w spec	3w cum	5w spec	5w cum	end	3w spec LOG	3w cum LOG	5w spec LOG	5w cum LOG	end LOG
mean	102.8	99.3	107.7	98.2	100.5	1.4	0.9	1.6	0.9	0.9
median	98.9	98.2	100.9	98.5	99.5	1.3	0.9	1.5	0.9	0.9
quantile .1	88.1	85.7	86.9	84.1	81.5	0.9	0.4	1.	0.5	0.5
quantile .9	120.	113.4	119.8	112.3	118.2	2.3	1.1	2.4	0.5	1.1
6	16	13	14	22						

peak +4	3w spec	3w cum	5w spec	5w cum	end	3w spec LOG	3w cum LOG	5w spec LOG	5w cum LOG	end LOG
mean	7484.1	789.6	7308.9	798.4	886.2	0.9	0.6	1.2	0.6	0.6
median	247.3	400.7	215.2	357.1	352.1	0.7	0.5	1.1	0.5	0.5
quantile .1	106.3	168.9	91.1	177.9	172.	0.4	0.4	0.6	0.4	0.4
quantile .9	1048.3	1145.7	1400.8	1026.2	956.6	1.6	0.8	2.1	0.4	0.8
45	39	36	40	42						

peak +8	3w spec	3w cum	5w spec	5w cum	end	3w spec LOG	3w cum LOG	5w spec LOG	5w cum LOG	end LOG
mean	205 220.	252 062.	205 950.	283 519.	306 522.	1.6	1.2	1.9	1.2	1.3
median	2797.2	3030.7	5405.2	3321.8	3947.1	1.4	1.1	1.6	1.2	1.2
quantile .1	205.3	418.5	105.8	362.7	270.3	0.7	0.5	1.	0.5	0.4
quantile .9	164 479.	21 833.4	864 440.	28 236.4	30 622.2	2.7	2.	3.2	0.5	2.1
29	6	29	5	7	{}	{}	{}	{}	{}	{}
